# How should the applications of genome editing be assessed and regulated?

**DOI:** 10.7554/eLife.26295

**Published:** 2017-04-04

**Authors:** Robin Fears, Volker ter Meulen

**Affiliations:** 1 Bioscience Programme at European Academies Science Advisory Council, Halle, Germany; 2 EASAC Working Group on Genome Editing and is at the German National Academy of Sciences Leopoldina, Halle, Germany

**Keywords:** science policy, regulation, EASAC, genome editing, CRISPR-Cas

## Abstract

An EASAC working group on genome editing recommends that regulators should focus on specific applications of these new techniques rather than attempting to regulate genome editing itself as a new technology.

Genome editing is helping to transform basic research in the life sciences and is expected to have applications in human and animal health, agriculture and food systems, and the bioeconomy. However, the regulatory status of these applications is still uncertain in some respects and discussions continue worldwide on how genome editing might be different from other biotechnologies in principle and in practice. In agriculture, for example, a decision on whether genome-edited plants should be considered as genetically modified organisms (GMOs) has been pending in the European Union for years and will now be considered by the European Court of Justice.

In order to help evaluate and clarify what is required to realise the opportunities presented by genome editing and to advise policy makers on options for governance and regulation, the European Academies Science Advisory Council (EASAC) constituted a Working Group containing the present authors and other scientists (see Acknowledgements) to prepare a report on the potential applications of genome editing and the related ethical and social questions. The EASAC report, endorsed by member academies, concentrated on issues of relevance for EU policy but these issues are of global importance (EASAC, 2017). Here we discuss some of the consensus conclusions and recommendations from the report. In our view, regulation must focus on the sector-specific applications rather than genome editing itself as a technology.

Genome editing, the deliberate alteration of a selected DNA sequence in a cell using site-specific nucleases, has become a vitally important tool in basic research to help understand biological functions and disease mechanisms. The first generation of genome-editing tools to achieve flexibility in target sequence recognition – zinc fingers nucleases and then transcription activator-like effector nucleases – appeared some 20 years ago. The field has since been revolutionised with the development of the CRISPR-Cas9 system (Jinek et al., 2012), which is comparatively much easier to design, produce and use. At the same time, research is continuing to identify and refine new genome-editing systems with even greater precision, reducing the likelihood of off-target effects, and to characterise other microbial sources of programmable nucleases to increase the repertoire of biotechnologies available (see, for example, Burstein et al., 2017).

Here we discuss genome editing with respect to the following areas: plant breeding in agriculture; animal research and development; gene drive based technologies; micro-organisms, the bioeconomy and biosecurity; and human health.

## Plant breeding in agriculture

There has been significant progress in the field of plant breeding over the past three decades, with a number of new molecular techniques being introduced. The increased precision now possible in plant breeding using genome editing technologies represents a big change from conventional breeding approaches that relied on random, uncontrolled chemical- or radiation-induced mutagenesis. Emerging opportunities in plant sciences include the simultaneous edits of multiple genes to increase crop quality by developing cultivars with improved water and nitrogen use efficiency, better resistance to pests and diseases, and reduced allergens (see, for example, Bortesi et al., 2016).

Research advances are being translated into novel crops. One of the first products to be allowed in the US is the CRISPR-Cas9-edited mushroom (which has reduced browning due to the decreased activity of polyphenol oxidase), but these scientific advances accentuate a continuing challenge for regulatory authorities worldwide. That is, to what extent should plant/food products developed using genome editing come within the purview of previous legislation governing GMOs? In our opinion, regulators should confirm that the products of genome editing, when they do not contain DNA from an unrelated organism, do not fall within the scope of GMO legislation. We also advise that while there should be full transparency in disclosing the process used, the aim should be to regulate the agricultural trait/product rather than the technology by which it is produced.

## Animal research and development

Research on animals is already subject to stringent regulation regarding animal health and welfare. It is also subject to the principles of the “3Rs” (replacement, reduction and refinement), with refinement perhaps being most relevant in this context. Although most genome-edited animals are currently being generated for basic or biomedical research purposes, there are also significant opportunities for livestock and aquaculture – to enhance animal health and welfare as well as to increase agricultural production. For example, recent research is conferring protection from porcine reproductive and respiratory syndrome, economically the most important disease affecting pigs in Europe, North America and Asia (Whitworth et al., 2016).

We recommend that livestock breeding should be governed by the same principles as proposed for crop plant breeding: to regulate the trait and not the technology, and to be open and explicit about what is being done. There is considerable laboratory research underway to develop cellular and animal, including larger animal, models of human disease. The concurrent multiple edits that are now feasible can help to reconstruct complex disease pathways in model organisms to identify and characterise therapeutic targets for functional screening. Additionally, it will be possible to introduce more predictive safety testing and new precision medicine regimens (Fellmann et al., 2017).

Genome editing is also helping to transform prospects in xeno-transplantation, by tackling barriers to the transfer of tissues and organs from animals to treat loss or dysfunction in patients (Perota et al., 2016). In particular, genome editing can remove the various xeno-reactive animal tissue epitopes, which would otherwise trigger both hyperacute and delayed rejection, and endogenous retroviruses. Also, as discussed in the EASAC report, genome editing is being explored in research on human-animal chimeras (for example growing a pig embryo with a human pancreas) to provide a source of human organs for transplantation. Editing is important here to inactivate specific human developmental genes to prevent any contribution to the chimera beyond the organ to be transplanted. The scientific community has an important continuing role in assisting regulators worldwide to prepare for the potential opportunities in transplantation now coming within range.

## Gene drive based technologies

Gene drive is a process of biased inheritance that enables a gene to be transmitted from parent to offspring at an increased rate. Such applications of genome editing, for insect disease vector control and other modifications of target populations, may offer significant potential to help address major public health challenges (Alphey, 2016). There are efficacy questions still to be answered by research. For example, to what extent does genetic diversity in natural populations provide a source of resistance to the gene drive? There are also safety considerations. If self-sustaining, will the spread of gene drive constructs have ecological consequences beyond those intended? What safeguards – physical, molecular and ecological – could be conceived to manage the risk of escape of a gene drive organism from laboratory research?

Recent recommendations by the US National Academies (National Academies, 2016) provide a sound framework for responsible development of the application. EASAC endorses their emphasis on the importance of a phased approach to research to allow sufficient time to evaluate the efficacy and safety of gene drives before regulatory decisions are made on whether or not they will be suitable for use. This phased research must include robust risk assessment and public engagement, including in those countries where gene-drive systems would most likely be applied to tackle disease vectors. The issues are of global relevance: a recent meeting of the UN Convention on Biological Diversity rejected calls for a moratorium on gene drives but encouraged caution in field testing and supported better risk assessment (Callaway, 2016).

Genome editing augments and extends the technologies previously available for the genetic alteration of microbes but, in our view, it does not raise significant new ethical or regulatory issues.

## Micro-organisms, the bioeconomy and biosecurity

Genome editing augments and extends the technologies previously available for the genetic alteration of microbes but, in our view, it does not raise significant new ethical or regulatory issues here. There is a wide range of potential applications in microbes (including biosynthesis of pharmaceuticals, other high-value chemicals and biofuels, and as biosensors), in bioremediation, and in the food chain. It is important for policy makers to recognise this wide range when developing strategies to support innovation in the bioeconomy.

Concerns have been expressed (Ledford, 2015) that microbial research may come to be conducted outside of the regulated laboratory setting, for example by the Do-It-Yourself biology community, but it has also been said that this community is active in developing responsible research norms (Kuiken, 2016). In other policy developments, it has been recommended, for example by the previous President’s Council of Advisors on Science and Technology in the US (PCAST, 2016) that misuse of technologies such as CRISPR must now be taken into account when setting biodefence strategy, to prepare for combatting state-sponsored terrorism or other misuse of the technology. Regulatory options are also being discussed as part of the ongoing scientific underpinning of the Biological and Toxin Weapons Convention. Later this year, EASAC together with other academies will convene a workshop to explore the nature and extent of the biosecurity implications of genome editing.

## Human health: somatic and germline cell applications

In addition to providing new tools for drug discovery, by establishing better screening approaches in vitro and in animal models, and by supporting new biosynthetic pathways to generate complex molecules, genome editing is being employed for novel gene- and cell-based therapies in healthcare (somatic cell editing) and could directly correct heritable disease-causing mutations in germline interventions (Fellmann et al., 2017). According to our working group and other similar initiatives (Academy of Medical Sciences, 2016; National Academies, 2017), it is vital for basic research to proceed intensively, subject to appropriate legal and ethical rules and standardised practices. In particular, according to the current rules, if early human embryos or germline cells undergo genome editing in the process of this research, the modified cells must not be used to establish a pregnancy. Proposals to use genome editing on human somatic cells for clinical research and treatment should be rigorously evaluated within the current regulatory frameworks for gene and cell therapies, with each case assessed for its potential benefits and risks, such as those that might arise from inaccurate editing.

These risks also apply when contemplating the application of genome editing to human germline cells, but there are many additional considerations to take into account when predicting potentially harmful effects. These include the obligation to consider the interests of the individual and future generations who will carry the genetic alteration, and the concern that the scope of applications might be widened beyond the prevention and treatment of disease to include biological enhancement. EASAC shares the view that it would not be acceptable to proceed to germline interventions unless and until the relevant scientific, ethical, safety and efficacy issues have been resolved and there is broad societal consensus.

Genome editing is being employed for novel gene- and cell-based therapies in healthcare and could directly correct heritable disease-causing mutations in germline interventions.

The latest recommendations from the US National Academies are ambitious in raising the prospect of identifying circumstances in which clinical research trials would be permissible for germline genome editing (National Academies, 2017). These circumstances would include a compelling clinical purpose and stringent oversight system. Notwithstanding these rigorous criteria, it is likely that such recommendations will continue to elicit controversy worldwide. While these matters are debated (The Lancet, 2017), it is vital to ensure that there is no general restriction on basic research and methodological development that would limit clarification of the technology’s potential.

## Realising the potential

In addition to the sector-specific elements for assessment and regulation, there are cross-cutting issues relevant to the progression of all of the applications of genome editing (and other emerging technologies in the biosciences). There has to be better engagement between scientists and the public-at-large as well as with policy makers. Scientists must continue to articulate the objectives for their research, the potential benefits, and the risk-management practices adopted. At the same time, additional social science and humanities research is warranted to underpin improved public engagement strategies. The scientific community must also work with others to ensure that an increasing uptake of these technologies does not accentuate inequity in access to the benefits. Furthermore, there is a responsibility to clarify to policy makers the broader consequences of their specific decisions about regulation and innovation. For example, previous decisions in the EU on genetic engineering technologies with respect to GMOs in agriculture have created difficulties for scientists, farmers and politicians in developing countries.

To reiterate, EASAC is of the view that policy considerations should focus on sector-specific applications rather than on genome editing itself as an emerging technology. It is important to avoid inadvertently constraining innovation. The assessment and regulation of applications must be evidence-based, it must take into account the likely benefits as well as any hypothetical risks, and it must be proportionate and sufficiently flexible to cope with future advances in the science.
